# Mental health during the covid-19 pandemic and domestic violence from the point of view of work at the institute of forensic psychiatry

**DOI:** 10.1192/j.eurpsy.2023.1130

**Published:** 2023-07-19

**Authors:** A. R. Bunjaku, G. Halilaj, G. Bunjaku, J. Lama

**Affiliations:** 1 Forensic Psychiatric Institut of Kosovo; 2 Life Diagnostico; 3 Anatomy Institut, Medicine Faculty Prishtina, Prishtina, Kosovo

## Abstract

**Introduction:**

The Institute of Forensic Psychiatry is an institution which, with the orders of the courts, evaluates the mental state of persons who have committed criminal offenses of all kinds.

From 2019, with the entry into force of the new penal code in Kosovo, even domestic violence is a criminale offense punishable by law up to three years in prison.

**Objectives:**

With the beginning of the pandemic and the measures that have been put into place by the government, which also meant the complete closure of many institutions and businesses with the aim of preventing the spread of the disease, restrictions on movement, and the presentation of many other problems such as the economy, the purchasing power, the loss of jobs that led to an increase in requests made by the courts to the Forensic Psychiatric Institute for the evaluation of the mental state of many perpetrators of domestic violence.

**Methods:**

Data were collected retrospectively from March 2019 to March 2020 in the time before the pandemic. March 2020 to March 2021 during the Lock Down, and March 2021 to March 2022, the time after the pandemic when we did not have these measures. These data have been provided by the archive of the Forensic Psychiatric Institute by collecting all the cases - the orders of the courts where the persons have been accused of the crime of domestic violence under Article 248 of the Criminal Code of the Republic of Kosovo.

**Results:**

During the Lock Down, there was an increase in cases of domestic violence. The number of requests from the Courts in the Forensic Psychiatric Institute increased from 494 before the pandemic to 648 orders during the pandemic and a slight decrease to 562 orders after the pandemic. The criminal offense with which they were accused most often was domestic violence from 119-23.68% of cases before the pandemic, in 202-3.17% of cases during the lockdown and a slight decrease after the pandemic in 156-27.75% of cases.

An increase in domestic violence caused by the female gender was also observed from 19 cases - 16.23% of all cases referred before the pandemic 61 cases - 30.19% of cases during the pandemic and a slight decrease in the time after repentance in 29 cases - 19.86%.

**Conclusions:**

During the pandemic, domestic violence experienced a significant increase that was a consequence of the government’s lock down measures to prevent the spread of the disease.

The number of cases of reoccurrence of violence in the family also increased among people who have had problems with mental health before.

There has also been a significant increase in domestic violence caused by the female gender, which was unexpected for our culture.

**Disclosure of Interest:**

None Declared

